# Supplementary material for: Sustained-input switches for transcription factors and microRNAs are central building blocks of eukaryotic gene circuits
Source: Genome Biol. 2013 Aug 23;14(8):R85. doi: 10.1186/gb-2013-14-8-r85 (PMC4054853; doi:10.1186/gb-2013-14-8-r85)
Supplement: Additional file 5 — HTML Browsable Motif Output. Zipped folder containing all WaRSwap and FANMOD motif output, viewable in a web browser. [file gb-2013-14-8-r85-S5.ZIP › HTML_browsable_motif_output/WaRSwap_ath_tair9/sigs_WSWRSLDraw_2-2000.pvals.heatmaps.html/motif_id_46_011101002_tftype_ath_upstream_-3000_0.html]

```
BG_MODEL = Rnew2
MOTIF_ID = 46_011101002
TF_TYPE = ath
UPSTREAM = -3000_0


PVals
FN_0.2	FN_0.4	FN_0.6	FN_0.8
dg_60.genes	0.054	0.021	0.004	0.775
dg_70.genes	0.059	0.021	0.005	0.77
dg_80.genes	0.051	0.025	0.002	0.751

ZScores
FN_0.2	FN_0.4	FN_0.6	FN_0.8
dg_60.genes	1.419	1.909	2.815	-0.855
dg_70.genes	1.405	2.02	2.925	-0.86
dg_80.genes	1.406	1.899	2.962	-0.85

StDevs
FN_0.2	FN_0.4	FN_0.6	FN_0.8
dg_60.genes	158132.483	80468.756	16883.139	1242.64
dg_70.genes	161413.742	78194.623	16584.554	1209.6
dg_80.genes	155482.074	82670.809	16379.857	1176.711
```
